# Discordance between *ABC* blood phenotype and genotype in a domestic short‐haired cat

**DOI:** 10.1111/jvim.16927

**Published:** 2023-11-03

**Authors:** Argyrios Ginoudis, Bertrand Canard, Alexandra Kehl, Urs Giger, Christos Koutinas, Stella Christoforaki, Erasmia Smyroglou, Zoe Polizopoulou, Mathios E. Mylonakis

**Affiliations:** ^1^ Diagnostic Laboratory, School of Veterinary Medicine, Faculty of Health Sciences Aristotle University of Thessaloniki Thessaloniki Greece; ^2^ Alvedia Limonest France; ^3^ Laboklin GmbH & Co. KG Bad Kissingen Germany; ^4^ Comparative Experimental Pathology, School of Medicine Technical University of Munich (TUM) Munich Germany; ^5^ Vetsuisse Faculty University of Zürich Zürich Switzerland; ^6^ Companion Animal Clinic, School of Veterinary Medicine, Faculty of Health Sciences Aristotle University of Thessaloniki Thessaloniki Greece

**Keywords:** alloantibodies, blood typing, Bombay phenotype, crossmatch, feline, H antigen

## Abstract

An adult domestic short‐haired feline leukemia virus‐infected cat was referred for kidney failure and worsening anemia requiring transfusions. *ABC* blood typing was performed with an immunochromatographic strip assay at different occasions. Gel column systems were used for the major and minor crossmatching tests, and anti‐*A* and anti‐*B* titers were determined. No discrete *A* or *B* bands appeared on the immunochromatographic strips at any time point for the recipient cat. The recipient's plasma agglutinated RBCs from tested type *A* and *B* cats. The recipient's RBCs appeared compatible with plasma from 1 type *A* and 2 *B* donors, and incompatible with plasma from another type *A* cat. Genotyping of recipient blood revealed a single homozygous c.179G>T *CMAH* variant predicting a blood type *B*. These studies suggest an unusual weak type *B* or missing all *ABC* antigens. The latter resembles the exceedingly rare Bombay phenotype in the human *ABO* blood group system.

**Abbreviations**
CMAH: cytidine monophosphate‐N‐acetyl‐neuraminic acid hydroxylase
FeLV: feline leukemia virus
FIV: feline immunodeficiency virus
HCT: hematocrit
ICS: immunochromatographic strip
NeuAc: N‐acetyl‐neuraminic acid (type *B* antigen)
NeuGc: N‐glycolyl‐neuraminic acid (type *A* antigen)
RBC: red blood cells
RI: reference interval

## 1 INTRODUCTION

The major feline blood antigen system characterizes cats as *A*, *B*, or *C* (also known as *AB*). All type *B* cats older than 3 months have naturally occurring anti‐*A* alloantibodies, triggering potentially life‐threatening acute hemolytic reactions and neonatal isoerythrolysis.^1^, ^2^ The prevalence of *ABC* blood types varies geographically and among breeds, with type *A* being the most common type.^3^, ^4^ The blood type antigenic determinants of the red blood cell (RBC) membrane are N‐glycolyl‐neuraminic acid (NeuGc) for type *A* and N‐acetyl‐neuraminic acid (NeuAc) for type *B*.^5^, ^6^ Blood types are determined by variants of the gene that encodes cytidine monophospho‐N‐acetylneuraminic acid hydroxylase (CMAH), the enzyme responsible for the conversion of NeuAc to NeuGc.^4^ As a result, type *B* cats express only NeuAc and type *A* cats possess predominantly NeuGc, whereas type *C* cats have approximately equal amounts of NeuAc and NeuGc on the RBC membrane.^3^

Bombay blood group phenotype, an exceptionally rare blood type in humans outside of Southeast Asia, occurs in approximately 1 in a million individuals in Europe. This blood type is characterized by the absence of the H antigen on RBCs. As the H antigen is the substrate on which the *A* and *B* antigens are formed, the individuals lacking this antigen are unable to produce *A* or *B* antigens and appear as type *O* on routine *ABO* phenotyping, regardless of their *ABO* genotype.^7^, ^8^, ^9^

In this case report, blood type studies that resemble the exceedingly rare Bombay phenotype or a weak type *B* are described in a domestic short‐haired cat.

## 2 CASE HISTORY

A 4.5‐year‐old neutered male domestic short‐haired cat was referred to the Companion Animal Clinic, School of Veterinary Medicine, Aristotle University of Thessaloniki, Greece. The cat was presented with a 3‐week history of worsening anorexia, lethargy, halitosis, and vomiting. Physical examination on admission revealed poor body condition (2/5), moderate pallor, lethargy, ulcerative lesions on the dorsal surface of the tongue, and a grade IV/VI left‐sided systolic murmur.

CBC (ADVIA 120 Hematology and Cytometry System, Bayer HealthCare LLC, Diagnostics Division, Tarrytown, New York, USA) and blood smear examination demonstrated a normochromic, normocytic, non‐regenerative (reticulocytes: 5400/μL; reference intervals [RI]: <80 000/μL) anemia (hematocrit [HCT] 16%; RI: 30%‐45%), neutrophilia (neutrophils: 29 400/μL; RI: 3000‐13 400/μL), and a moderate thrombocytopenia (102 000/μL; RI: 300 000‐800 000/μL), in the presence of a few platelet aggregates on the blood smear. A serum biochemistry panel (Vitalab Flexor E Biochemistry Analyzer, Vital Scientific NV, Dieren, Netherlands) indicated increased concentration of creatinine (6.1 mg/dL [RI: 0.7‐1.6 mg/dL]), urea nitrogen (117 mg/dL [RI: 9‐32 mg/dL]), and inorganic phosphorus (10.1 mg/dL [RI: 3.5‐6.7 mg/dL]). Urinalysis showed isosthenuria (1.012; crystalloids had been given before admission), a urinary protein‐to‐creatinine ratio of 1.8 (RI < 0.4), and an inactive urinary sediment; urine culture failed to grow bacteria. The arterial blood pressure was normal. Serology was positive for feline leukemia virus (FeLV) antigen and negative for feline immunodeficiency virus (FIV) antibodies using an in‐office ELISA test (Feline SNAP Combo Plus, IDEXX Laboratories Inc., Maine, USA). Abdominal ultrasonography indicated increased cortical thickness and poorly defined corticomedullary junction of the left kidney and a normally appearing right kidney.

**FIGURE 1:**
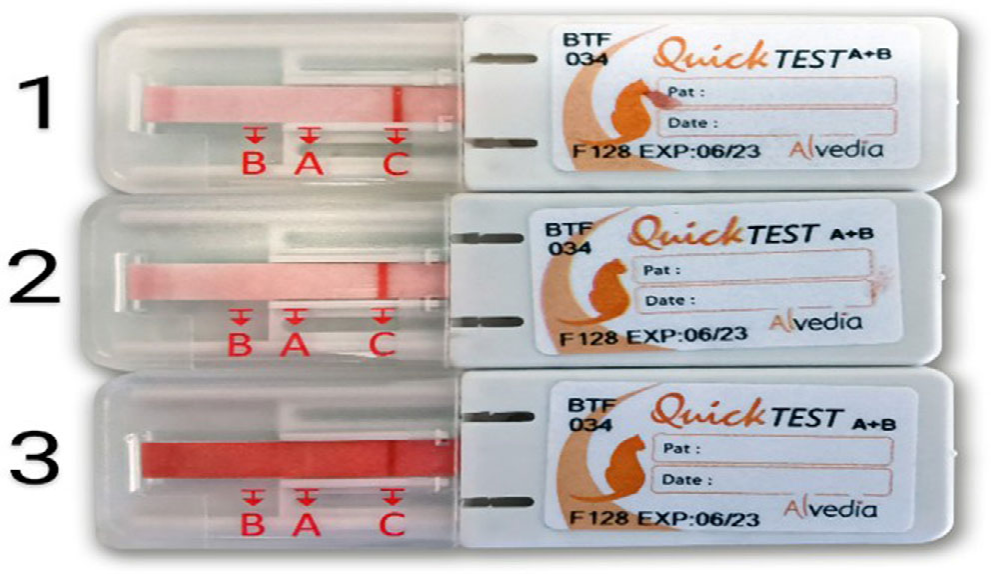
In‐clinic blood typing with the immunochromatographic strip using the cat's whole blood (1, 2) and concentrated red blood cells after decanting the plasma (3). The control band is clearly seen, as opposed to no discrete *A* or *B* bands. Type *O* in the feline *ABC* system has not previously been described in cats.

**FIGURE 2:**
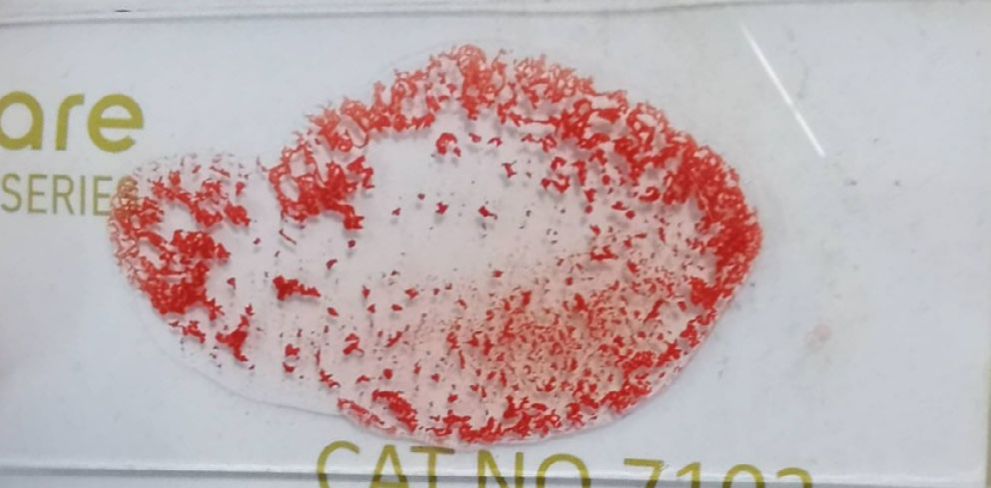
Glass slide back typing by mixing 2 drops of recipient serum and 1 drop of reference *A* red blood cells. Gross agglutination is seen.

Due to the severe, likely chronic kidney disease (international renal society‐based stage IV, with proteinuria), the cat was hospitalized. Serial CBC results indicated worsening of anemia (HCT: 13% on third day post‐admission) requiring a transfusion. Blood typing was performed by an in‐clinic immunochromatographic strip (ICS) assay (Feline Quick Test BT, Alvedia, Limonest, France). Unexpectedly, no discrete *A* or *B* bands appeared on the strip, whereas the control band was clearly visible (Figure 1). Blood typing was attempted 2 more times with the same ICS assay, including 1 with concentrated RBCs after whole blood centrifugation and decanting of the plasma showing the same results (Figure 1). In addition, a glass slide back typing was performed by admixing 2 drops of the patient's serum and 1 drop of reference type *A* packed RBCs (BSA Animal Blood Bank, Porto, Portugal).^3^ Gross agglutination was noticed (Figure 2), implying that recipient's blood was more likely type *B*. Since a type *B* blood unit was not accessible at the time, no blood was transfused and the cat was treated with recombinant human erythropoietin A, along with isotonic fluids and other supportive treatments. The cat's clinical condition and clinicopathologic parameters exacerbated, which prompted the owner's decision for humane euthanasia 1‐month after admission. No permission for necropsy was granted.

**FIGURE 3:**
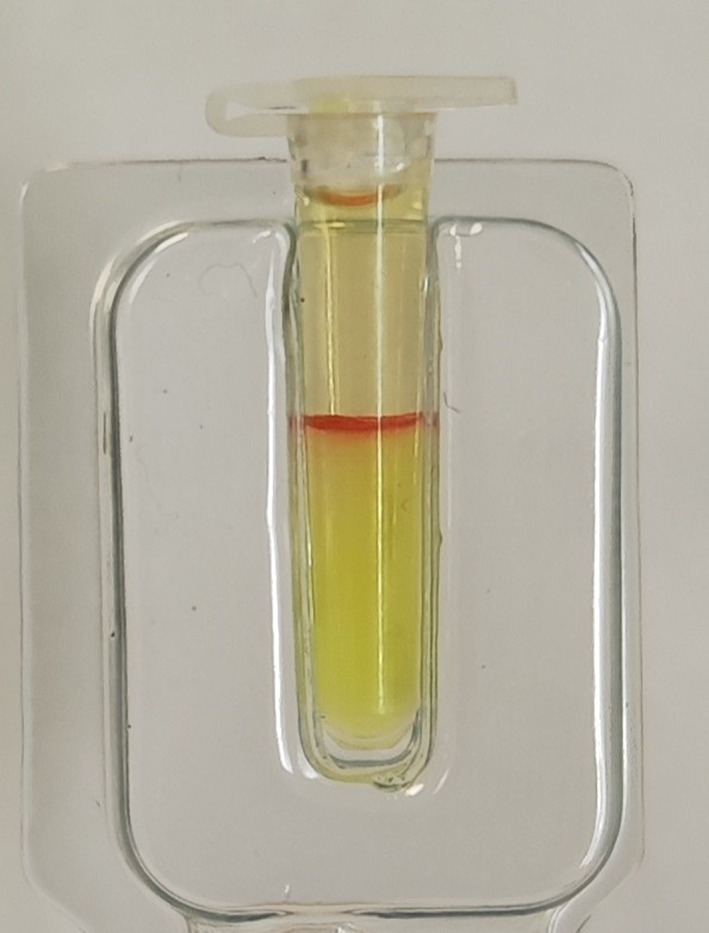
Major crossmatching test with the cat's plasma and type *A* (2 cats) or type *B* (2 cats) red blood cells. Identical agglutination pattern as indicated here was noticed in all instances.

**FIGURE 4:**
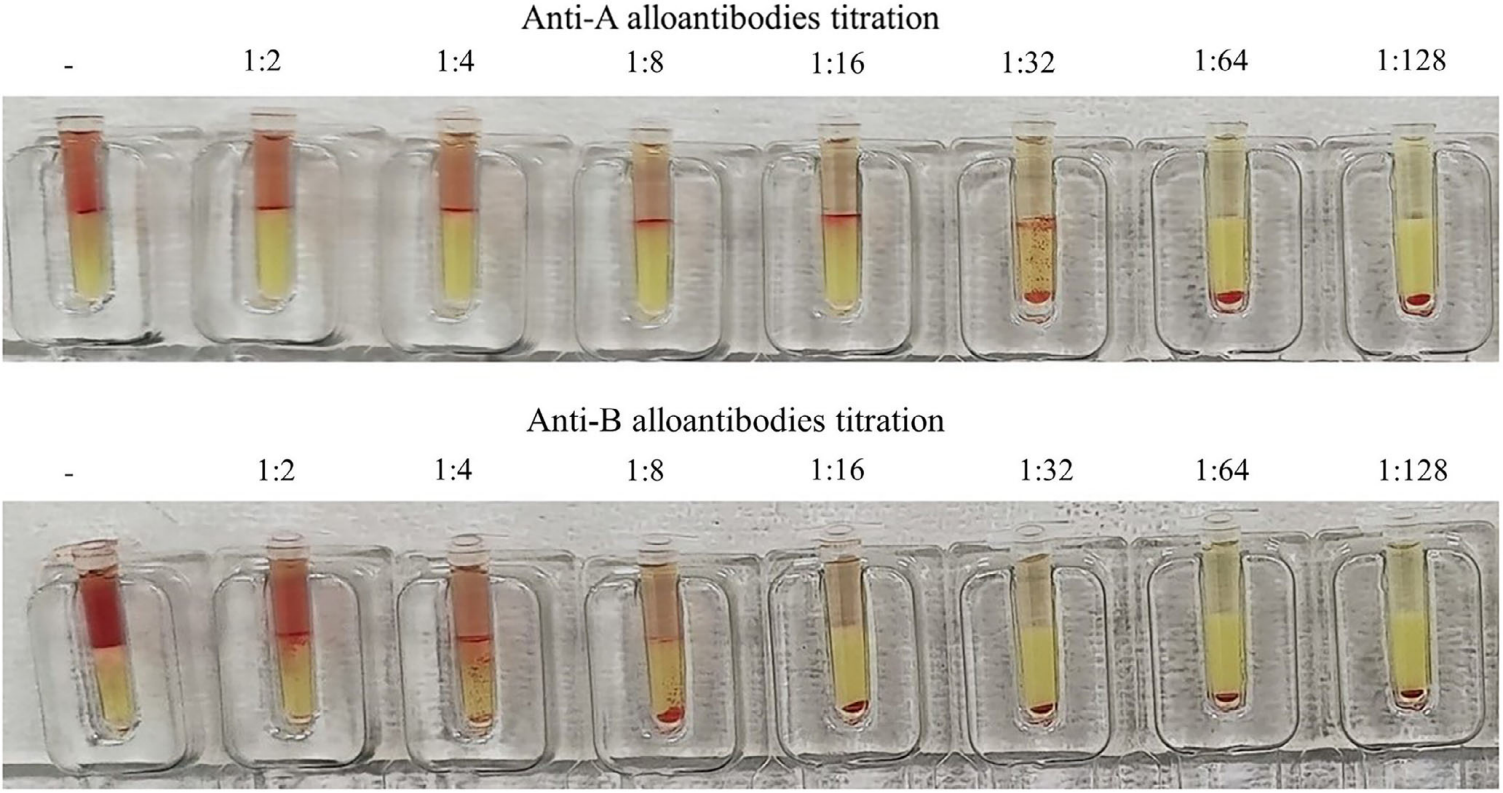
*Upper part*: Titration of anti‐*A* alloantibodies using crossmatching tests with consecutive dilutions of the cat's plasma and red blood cells (RBCs) from 2 type *A* cats. A positive crossmatch was noticed until dilution 1/32 in both cats. *Lower part*: Titration of anti‐*B* alloantibodies using crossmatching test with consecutive dilutions of the cat's plasma and RBCs from 2 type *B* cats. A positive crossmatch was noticed until dilution 1/4 in both cats.

**FIGURE 5:**
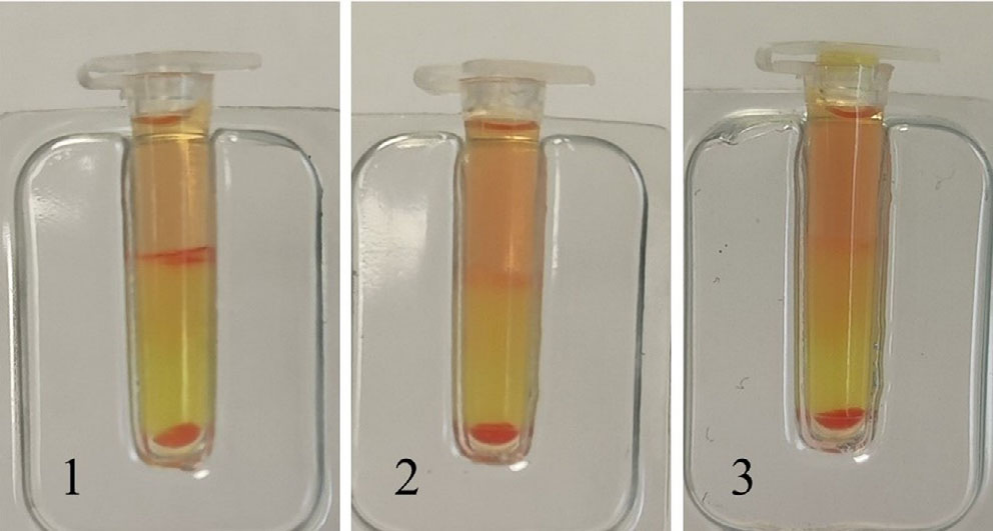
Minor crossmatching test using the cat's red blood cells and plasma from a type *A* cat, indicating a positive (incompatible) crossmatch (1). A negative (compatible) crossmatch was seen with another *A* cat (2) and the plasma from 2 type *B* cats (3).

Due to the unexpected blood typing results, 2 left‐over EDTA‐anticoagulated blood samples were sent to the manufacturer of the ICS feline typing assay for further assessment. Cartridges from a different ICS lot failed anew to type as *A* or *B*. Based upon major and minor crossmatching test performed using a gel column test (Feline Gel Test XM, Alvedia, Limonest, France), the recipient's plasma was crossmatched against RBC samples from 2 type *A* and 2 type *B* donor cats, resulting in agglutination in all instances (Figure 3). Titration of anti‐*A* and anti‐*B* alloantibodies, mixing the recipient's plasma against RBCs from 2 *A* and 2 *B* donor cats, different from those used in the major crossmatching, indicated a positive reaction up to a dilution of 1/32 against both *A* donor RBCs, and up to a dilution 1/4 against both *B* donor RBCs (Figure 4). A negative (compatible) crossmatch was found when the plasma samples from 2 type *B* cats were tested against RBCs from the recipient; however, whereas a negative (compatible) crossmatch was observed with plasma from 1 type *A* cat, a positive (incompatible) crossmatch was noticed with the plasma of the second type *A* cat (no titration available; Figure 5).

A genotyping panel of 4 *CMAH* variants was assessed using frozen EDTA‐anticoagulated blood, which revealed a single homozygous c.179G>T *CMAH* variant predicting a type *B*.^10^

## 3 DISCUSSION

This is a report of a cat without appreciable *A* or *B* antigen on erythrocytes assessed by an in‐clinic typing kit. The cat described herein was confirmed as of *bb* genotype, bearing the *CMAH* variant c.179G>T, which results in blood type *B* in Turkish domestic cats and other breeds.^10^, ^11^ As the home of this domestic short‐haired cat is Greece and neighboring Turkey, it is not surprising to find the same *CMAH* variant. However, this does not explain the cat's phenotype, as an ICS assay failed multiple times to identify the cat's blood type as *A*, *B*, or *C* (*AB*), including 1 time using concentrated RBCs, suggesting a “null” phenotype (ie, both NeuAc and NeuGc were missing), in the context of detectable anti‐*A* (titer 1/32) and anti‐*B* (titer 1/4) alloantibodies. The fact that NeuAc appears not absorbed or synthesized and at the same time the CMAH enzyme is also mutated, might indicate that *CMAH* is further changed to a precursor antigen different than NeuAc or NeuGc. Overall, these blood type studies bear similarities with an unusual “weak *B*” phenotype or the exceedingly rare Bombay‐like phenotype of the human *ABO* blood group system, although a precursor substrate analogous to the H antigen in the *ABO* system has yet to be identified in cats.^8^, ^9^

Humans with the Bombay phenotype appear to type as group *O* by the *ABO* testing, regardless of their *ABO* genotype, as they lack the H‐antigen scaffold on RBCs to form the *A* and *B* antigen.^7^ Therefore, when anti‐*A* and anti‐*B* reagents are matched against their RBCs, no agglutination is seen, but they produce antibodies against the *A* and *B* RBC antigens, as well as express a clinically important anti‐H titer. Individuals with the Bombay phenotype must only receive H antigen‐negative blood.^9^ In this cat, testing against the precursor substrate was technically not possible.

Uncommon human blood type variants in the *ABO* system, are weak *A* and *B* subgroups. Individuals with these phenotypes possess decreased *A* or *B* antigens on their RBCs and show variable degrees of agglutination with anti‐*A* or anti‐*B* antibodies, respectively. It is suggested that weak blood group *B* phenotypes might be caused by sequence variations in the CBF/NF‐Y regulatory region of the *ABO* gene.^12^ A low percentage of individuals with weak *A* or *B* subgroups contain anti‐*A* or anti‐*B* antibodies in their plasmas as observed in the cat of this report.^13^

Blood type genotype‐phenotype discordance are rarely reported in cats, although earlier reports were not identical with this case.^10^, ^11^, ^14^ Recently, 5 distinct RBC antigens beyond the *ABC* blood group system were hypothesized to be present and 1 of these was thought to correspond to the previously described Mik antigen.^15^, ^16^ Importantly, the results of blood typing might uncommonly be affected by disease states in humans and animals. For example, loss of *A* and *B* antigens from the RBCs of human patients with hematological malignancies is a fairly frequent occurrence and DNA methylation of the *ABO* promoter was shown to account for the loss of *ABO* allelic expression in leukemic patients.^17^ In a study of cats, 4 blood type *A* domestic short‐haired cats with FeLV‐related anemia were mistyped as *AB* type, even after eliminating by washing the weak autoagglutination in 3 of these cats.^18^ Some likely form of antigenic mimicry with the *B* antigen on the surface of RBCs or a reduction in CMAH activity converting NeuAc to NeuGc in type *A* cats might explain these results. The cat of this report was FeLV positive; it remains unknown if this affected the antigen expression and alloantibody titers. The advanced chronic kidney disease prompted the owner's decision for euthanasia 1‐month post‐admission, not allowing to retype the cat in the long term to check for possible phenotype reversal. In a recent multicenter study, no association between overall retroviral status (FeLV or FIV positive/negative) and blood type, between FeLV status and blood type or between FIV status and blood type was established and no difference was found in the distribution of blood types between healthy or sick cats.^19^ In the latter study, the number of FeLV‐antigenemic cats was relatively small (n = 47), which might have adversely affected the validity of the results.

Historically, the ICS assay used in this cat has reliably detected the *ABC* blood types in anemic and non‐anemic cats. In 1 study, its diagnostic sensitivity was found to be 97.7% and 95.7% for blood type *A* and *B*, respectively, whereas its specificity was 100% for *A* and 97.1% for *B* blood type, respectively, whereas a second study found a sensitivity and specificity of 100% for all blood types.^20^ Unfortunately, because of the owner's decision for euthanasia, it was not technically feasible in this case to collect more fresh blood and repeat the blood typing by alternative methods, such as the historically considered gold‐standard tube method, gel column or flow cytometry, or other reagents (ie, *Triticum vulgaris* lectin).^3^, ^18^, ^20^, ^21^, ^22^

In conclusion, in this domestic short‐haired cat, the constellation of the inability to establish the cat's *ABC* blood group by an ICS assay, the back typing result, the major/minor cross matching results, along with the genotyping results, resemble the exceedingly rare Bombay phenotype in the human *ABO* blood group, although a substrate antigen analogous to the H antigen has not been yet identified in cats. The possibility of a weak type *B* similar to human medicine, with anti‐*A* and anti‐*B* antibodies and low *B* antigen site density on the cat's RBCs, which makes them undetectable with the common typing kits cannot be excluded.

## CONFLICT OF INTEREST DECLARATION

B. Canard works for Alvedia, A. Kehl works for Laboklin GmbH & Co. and U. Giger has been a scientific advisor to Alvedia and Laboklin. No other authors declare a conflict of interest.

## OFF‐LABEL ANTIMICROBIAL DECLARATION

Authors declare no off‐label use of antimicrobials.

## INSTITUTIONAL ANIMAL CARE AND USE COMMITTEE (IACUC) OR OTHER APPROVAL DECLARATION

Authors declare no IACUC or other approval was needed.

## HUMAN ETHICS APPROVAL DECLARATION

Authors declare human ethics approval was not needed for this study.
